# Sharenting: hidden pitfalls of a new increasing trend– suggestions on an appropriate use of social media

**DOI:** 10.1186/s13052-024-01584-2

**Published:** 2024-01-25

**Authors:** Antonio Gatto, Antonio Corsello, Pietro Ferrara

**Affiliations:** 1 Institute of Pediatrics, Catholic University, Roma, Italy; 2 Department of Clinical Sciences and Community Health, University of Milan, Milan, Italy; 3 Department of Medicine and Surgery, Università Campus Bio-Medico, Roma, Italy; 4 Operative Research Unit of Pediatrics, Fondazione Policlinico Universitario Campus Bio-Medico, Roma, Italy

**Keywords:** Sharenting, Parental management, Children’s rights, Multimedia, Education, Digital awareness

## Abstract

**Background:**

The term “sharenting”, defining the practice of sharing children’s photos on social media, has become widespread globally. This phenomenon introduces new risks for children, often overlooked by parents lacking experience or caution in protecting their children from potential harms.

**Main body:**

Parents share multimedia contents with positive intentions, but the lack of immediate risk perception prevails. An Italian study revealed that a significant percentage of parents (68%) frequently share their children’s photos on social platforms, often without considering potential risks. Pediatricians play a crucial role in raising awareness among parents regarding the dangers associated with online sharing and must empower families with defensive strategies to safeguard children’s privacy.

**Conclusions:**

The commentary emphasizes the need for increased parental assistance in comprehending the risks of sharenting and using social media prudently. Pediatricians are pivotal in guiding parents, striking a balance between the natural urge to share children’s progress and an awareness of associated risks. Immediate action by scientific societies involves training and informing parents through various digital and print resources. A concrete regulation of this phenomenon is needed to protect children’s rights, but prioritizing digital awareness and education seems pivotal in mitigating sharenting-related risks.

## Background

Introduced in the Oxford English Dictionary in 2022 and now widespread throughout the world, “sharenting” defines the habit of sharing on social media photos of their children. The term comes from the union of share (to share) and parenting (to be parents). Steven Leckhart used this word for the first time in an article titled “The Facebook-Free Baby. Are you a mom or dad who’s guilty of ‘oversharing’? The cure may be to not share at all”.

Digital sharing introduces new, underexplored risks for children, and some parents may lack the necessary experience or caution to protect children from these harms [1]. However, in most cases, the intentions of parents who share photos of their offspring online are harmless: document the growth, share anxieties and worries, searching for emotional support, seeking information in educational, pediatric and school fields. Three types of circumstances are mostly published: daily life (the child who sleeps, plays, eats), outings or trips and special moments (Christmas, baptism, first day of school, birthdays). Bartholomew et al., evaluating the context of social media used by new parents, showed in their study that 98% of mothers and 89% of fathers affirmed that they uploaded photographs of their child to Facebook [2]. Of these, 93% of mothers and 71% of fathers reported it was “very likely” to “likely” that the photographs would be acknowledged.

The scope of this commentary is to highlight the growing habit to share videos, photos, and others information about children by parents and caregivers, placing particular attention on the need for regulation of this phenomenon, which involves a part of the population whose rights are easily violated. The role of pediatricians and scientific societies, in this dangerous and insidious social trend, should be to provide guidance and support to parents and children.

## Main text

### Italian data and legislative considerations

An Italian study, published at the end of 2017, demonstrated that 68% of the parents interviewed publish photos of their children on social profiles with a certain frequency, while 30% tend to publish them not only on their message boards, but also on Facebook groups or other less virtual spaces, checked and filtered according to the personal profile [1]. The 88% of mothers who publish photos of their children declared that they had set the privacy options in order to limit the circle of people who can view the content. In 83% of cases this meant selecting the “Friends” privacy option, but it does not seem like a significant limitation. In Italy the phenomenon seems more widespread for children from 0 to 3 years, whose images are shared by 86% of parents, and tends to decline with age, with 68% of parents admitting to sharing images of their children after the fourth year of age [1]. Considering the frequency of publication of photos in the sample considered, during a period of four weeks, 62% of parents publish at least 1 to 4 photos of their children [1].

To clarify the dimension of the phenomenon, it is demonstrated that, on average, a 5-year-old child will have appeared in a thousand photos posted publicly by their parents, almost 20 per year.

In our legislative system, the personal image is protected by various rules: the law on copyright which provides that no person’s portrait can be exhibited without the subject’s consent; Article 10 of the civil code, which allows the request for removal of an image that harms the dignity of a subject with the consequent possibility of compensation for damages. However, an ambiguity in the regulations that protect the image should also be highlighted as we speak of ‘subject consent’ which, in the case of a minor, must be offered by his legal guardian (Article 316 of the Italian Civil Code), i.e. the parents”.

Regarding sharenting, in Italy, there are already sentences that have ruled in favor of children who, at the age of majority, denounced their parents for the numerous images posted without their consent. In particular some court sentences have decreed that posting photos of children is a “complete violation” of the “protection of the image”, contemplated by Article 10 of the Italian Civil Code, of the “protection of the confidentiality of personal data”, provided for by the Italian Privacy Code, as well as the New York Convention where it establishes that “no child shall be subjected to arbitrary interference with his privacy, family, home or correspondence, nor to unlawful attacks on his honor and reputation” and that “the child has the right to the protection of the law against such interference or such affronts”.

Furthermore, Article 16 of the 1989 Convention on the Rights of the Child strengthens the protection of minors: “No child shall be subjected to arbitrary or unlawful interference with his or her privacy, family, home or correspondence nor to unlawful attacks on his or her honor and reputation. The child has the right to the protection of the law against such interference or attacks” [2–5]. To date, according to the Italian Privacy Code and the sentences just cited, the age for digital consent is set at 14 years.

In Italy, the Authority for Children’s Rights had already delivered a report in May 2022 to the Minister of Justice, with numerous suggestions on the age of access, baby influencers, and in particular, about the sharenting, it was proposed to extend to this phenomenon the rule already contained in the law on cyberbullying which allows minors to obtain the removal of their images.

### Social aspects and risks

It is important to highlight that an increasing number of minors connects to the internet every day, uses social networks or apps that steal their data with the help of the internet, resembling parents’ behavior [2, 3]. According to Save the Children, 54% of children between 6 and 10 years old use the connection from home as do 94% of teenagers [2]. The implications are various and equally relevant: from issues related to privacy and copyright of the images, up to the range of risks that can be encountered online and the failure to protect the minor’s rights established with the 1989 New York Convention.

The phenomenon of sharenting is a source of intense debate. In fact, parents who, for various psychosocial reasons, post their children’s multimedia content online expose themselves to the judgment of users of social networks. Generally, mothers and fathers share photos and videos with the positive purpose of sharing significant family experiences and milestones, but they often have no immediate perception of any risk. A recent Italian report found that online presence before 2 years of age is documented in 81% of children living in Western countries, 92% in the US and 73% in Europe [6]. The same Authors affirm that within a few weeks of birth, 33% of children have their photos and information posted online, while more than 30% of mothers regularly post photos of their children or images of ultrasound scans even during pregnancy [6]. Most studies showed sharenting concerns mainly women, especially in its broader sense of sharing representations of parenting and children. This should not be taken completely for granted, since some studies also documented fathers’ involvement [7]. Furthermore, very few papers reported on the experiences of other actors, external to the nuclear family such as grandparents engaging in “grand-sharenting” [8]. Even teachers represent an interesting and less explored area of inquiry for research to look at, especially in consideration of the normalization of children’s social media presence that may lead people to feel legitimized to create a digital identity for minors [9, 10].

As for “what” parents share, the range of daily living circumstances included in the literature is wide and includes narratives of pregnancy, ultrasound scans, breastfeeding images, photos and videos of the offspring published on several social media, but also written threads on parenting forums about various experiences, as well as blog posts and YouTube videos [10]. Besides, some Authors highlight that sons are more often mentioned than daughters in posts, whose contents reproduce gender stereotypes about clothing, poses and games. Some of these portraits may be embarrassing for children, because they represent intimate and private moments [11]. As for influencer and celebrity parents, posts often sponsor particular brands through moments of daily life, in order to create a semblance of relatedness and closeness with their followers [12].

In this setting it is clear that parents ignore the serious risks to which their children are subjected such as actual and future emotional distress, sexual exploitation, and in particular identity theft. The main forms of digital fraud are identity cloning, financial identity theft, criminal identity theft, synthetic identity theft, medical identity theft.

To evaluate the risk related to the improper use of internet, in Italy during 2021 there were 5316 cases of child pornography handled by the Postal Police, with an increase of 47% compared to the previous year, 3243 cases. The number of minors approached online by abusive adults is also growing, the majority under the age of 13, but there are also growing cases of online solicitation of children aged 0–9 [13, 14].

On this topic, in recent literature, there is a growing interest, and Romero-Rodríguez et al. in 2022 proposed the Validation of the Sharenting Evaluation Scale (SES). This scale was composed of 17 items based on three factors: implications, social behaviour, and self-control resulting in a reliable instrument to evaluate the adult’s sharenting [15].

Lastly, Table 1 reports statements outlined by the Italian Paediatrics Society concerning ‘Sharenting’. These five crucial suggestions serve as a blueprint for parents navigating the landscape of sharing their children’s content online. Emphasizing the profound impact of digital sharing on a child’s privacy, the Society underscores the necessity for prudence and caution while engaging in this prevalent practice. From cultivating awareness about building a child’s digital identity without explicit consent to safeguarding against identity theft risks, the guidelines delineate the crucial steps in fostering a secure online environment for children.

**Table 1:** The 5 suggestions of the Italian paediatrics society regarding sharenting

1. Be aware that sharenting is an increasingly widespread practice, but this does not mean that its potential dangers should be underestimated. Sharing images, videos and any type of content featuring children means, in fact, building a child’s “digital dossier” without her consent and without his knowledge.
2. Sharing materials and information regarding your children on social media must include a certain caution and, on many occasions, anonymity, because what is shared in a detailed and personal manner, such as the location or full name, could dangerously expose children to a series of risks, first and foremost identity theft.
3. Don’t share images of your children in any state of nudity. These images should always remain private due to the potential risk that they may be misused by others.
4. Enable notifications that alert parents when their child’s name appears in search engines.
5. Respect minors’ consent and right to privacy, then familiarize yourself with the policy relating to the privacy of the sites on which content is shared.

## Conclusions

There is an insufficient assistance for parents in understanding the potential risks of sharenting and using social media correctly. Pediatricians are central figures in boosting parents’ awareness in terms of dangers associated with online sharing. To protect children’s privacy, families ought to be empowered about possible defensive strategies. It is important to support mothers and fathers, balancing the natural inclination to proudly share their children’s progress with the awareness of the risks associated with the practice of sharenting.

In the immediate future, the mission of pediatric and non-pediatric scientific societies on this topic, must be to give a rapid and effective response to this phenomenon in terms of training and information for parents through digital and paper documents.

Certainly, a new regulation of this phenomenon could help to protect children’s rights, but the solution could be a greater digital awareness and improved education.

## Data Availability

Not applicable.
